# Iranian clinical practice guideline for amyotrophic lateral sclerosis

**DOI:** 10.3389/fneur.2023.1154579

**Published:** 2023-06-02

**Authors:** Reza Boostani, Nahid Olfati, Hosein Shamshiri, Zanireh Salimi, Farzad Fatehi, Seyed Arya Hedjazi, Atefeh Fakharian, Majid Ghasemi, Ali Asghar Okhovat, Keivan Basiri, Bahram Haghi Ashtiani, Behnaz Ansari, Gholam Reza Raissi, Seyed Ahmadreza Khatoonabadi, Payam Sarraf, Sara Movahed, Akram Panahi, Bentolhoda Ziaadini, Mohammad Yazdchi, Jalal Bakhtiyari, Shahriar Nafissi

**Affiliations:** ^1^Department of Neurology, Faculty of Medicine, Mashhad University of Medical Sciences, Mashhad, Iran; ^2^Department of Neurology, Faculty of Medicine, Tehran University of Medical Sciences, Tehran, Iran; ^3^Neuromuscular Research Center, Tehran University of Medical Sciences, Tehran, Iran; ^4^Psychiatry and Behavioral Sciences Research Center, Mashhad University of Medical Sciences, Mashhad, Iran; ^5^Department of Psychiatry, Faculty of Medicine, Mashhad University of Medical Sciences, Mashhad, Iran; ^6^Legal Medicine Research Center, Legal Medicine Organization, Tehran, Iran; ^7^Pulmonary Rehabilitation Research Center (PRRC), Shahid Beheshti University of Medical Sciences, Tehran, Iran; ^8^National Research Institute of Tuberculosis and Lung Diseases (NRITLD), Shahid Beheshti University of Medical Sciences, Tehran, Iran; ^9^Department of Internal Medicine, Faculty of Medicine, Shahid Beheshti University of Medical Sciences, Tehran, Iran; ^10^Department of Neurology, Faculty of Medicine, Isfahan University of Medical Sciences, Isfahan, Iran; ^11^Isfahan Neuroscience Research Center, Isfahan University of Medical Sciences, Isfahan, Iran; ^12^Department of Neurology, Faculty of Medicine, Iran University of Medical Sciences, Tehran, Iran; ^13^AL Zahra Research Institute, Isfahan University of Medical Sciences, Isfahan, Iran; ^14^Department of Physical Medicine and Rehabilitation, Faculty of Medicine, Iran University of Medical Sciences, Tehran, Iran; ^15^Neuromusculoskeletal Research Center, Iran University of Medical Sciences, Tehran, Iran; ^16^Department of Speech Therapy, School of Rehabilitation, Tehran University of Medical Sciences, Tehran, Iran; ^17^Iranian Center of Neurological Research, Neuroscience Institute, Tehran University of Medical Sciences, Tehran, Iran; ^18^Department of Neurology, Imam Khomeini Hospital Complex, Tehran University of Medical Sciences, Tehran, Iran; ^19^Department of Nutrition, Faculty of Medicine, Mashhad University of Medical Sciences, Mashhad, Iran; ^20^Metabolic Syndrome Research Center, Mashhad University of Medical Sciences, Mashhad, Iran; ^21^Department of Neurology, Faculty of Medicine, Kerman University of Medical Sciences, Kerman, Iran; ^22^Neurology Research Center, Kerman University of Medical Sciences, Kerman, Iran; ^23^Department of Neurology, Faculty of Medicine, Tabriz University of Medical Sciences, Tabriz, Iran; ^24^Neurosciences Research Center, Tabriz University of Medical Sciences, Tabriz, Iran

**Keywords:** amyotrophic lateral sclerosis, guideline, multidisciplinary (care or team), edaravone, physical therapy, nutrition, mechanical ventilation, genetic testing

## Abstract

Amyotrophic lateral sclerosis (ALS) is a rapidly progressive neurodegeneration involving motor neurons. The 3–5 years that patients have to live is marked by day-to-day loss of motor and sometimes cognitive abilities. Enormous amounts of healthcare services and resources are necessary to support patients and their caregivers during this relatively short but burdensome journey. Organization and management of these resources need to best meet patients' expectations and health system efficiency mandates. This can only occur in the setting of multidisciplinary ALS clinics which are known as the gold standard of ALS care worldwide. To introduce this standard to the care of Iranian ALS patients, which is an inevitable quality milestone, a national ALS clinical practice guideline is the necessary first step. The National ALS guideline will serve as the knowledge base for the development of local clinical pathways to guide patient journeys in multidisciplinary ALS clinics. To this end, we gathered a team of national neuromuscular experts as well as experts in related specialties necessary for delivering multidisciplinary care to ALS patients to develop the Iranian ALS clinical practice guideline. Clinical questions were prepared in the Patient, Intervention, Comparison, and Outcome (PICO) format to serve as a guide for the literature search. Considering the lack of adequate national/local studies at this time, a consensus-based approach was taken to evaluate the quality of the retrieved evidence and summarize recommendations.

## 1. Introduction

Amyotrophic lateral sclerosis (ALS) is a rapidly progressive neurodegenerative disorder of the motor neurons. The site of onset is variable in ALS based on the phenotype, although a spinal/limb onset (58–82%) followed by bulbar onset (28%) is the classical phenotype (1). The spinal onset phenotype is further classified into typical spinal onset in which lower motor neuron (LMN) and upper motor neuron (UMN) symptoms start in one limb and rapidly spread to all other limbs, bulbar, and thoracic regions; flail arm and leg phenotypes in which predominantly LMN symptoms remain restricted to the upper or lower limbs, respectively, for at least 12 months; hemiplegic in which predominantly UMN symptoms remain restricted to ipsilateral upper and lower limbs; and psudopolyneuritic phenotype presenting with distal-predominant LMN findings. Other rare phenotypes include progressive muscular atrophy (PMA), primary lateral sclerosis (PLS), mixed, and thoracic/respiratory onset (2).

The short life of ALS patients is burdened by progressive loss of motor and sometimes cognitive abilities. Therefore, an enormous amount of healthcare services and resources are vital throughout this journey to support patients and their caregivers. These include but are not limited to regular neurologic evaluations, speech therapy, physical and occupational therapy, pulmonary function evaluation and interventions, nutritional interventions and enteral feeding, psychiatric evaluations, and palliative care. The worldwide solution that allows integrated care for these patients while also preventing escalated healthcare costs is multidisciplinary ALS clinics which are known as the gold standard of ALS care. It is a quality milestone for the Iranian healthcare system to introduce this standard to the care of Iranian ALS patients. To this end, a national ALS clinical practice guideline is the necessary first step.

## 2. Methods

A team of national neurology, neuromuscular, rehabilitation, pulmonology, speech-language pathology, psychiatry, legal and medical ethics, and nutrition experts participated to develop the Iranian ALS clinical practice guideline. Clinical questions were formulated in the Patient, Intervention, Comparison, and Outcome (PICO) format to guide the literature search. PICO questions were finalized in a panel discussion. Search strategies were developed based on the PICO questions, and relevant literature was retrieved from Medline, Embase, Cochrane Database of Systematic Reviews (CDSR), and Cochrane Central Register of Controlled Trials (CENTRAL) filtering for English and Persian language. Considering the current lack of adequate national/local studies, a consensus-based approach was taken to evaluate the quality of the retrieved evidence and formulate recommendations. Panel discussions were held to present the findings of the literature search and draft recommendations. The recommendations were finalized in separate panel discussions. We graded the strength of the recommendations based on experts' consensus on scientific rigor and quality of evidence, as well as feasibility measures including availability, cultural acceptance, specific legal aspects, and relevant costs. Grade A was applied when a highly feasible recommendation was supported by high-quality evidence, and grade B was applied when feasibility was lower or lower-quality evidence was available for a highly feasible recommendation. Grade C was applied when both evidence quality and feasibility were low, and grade E was applied to good clinical practice points solely based on expert opinion with any feasibility rating. The final guideline was reviewed and approved by all experts.

## 3. Results

### 3.1. Epidemiology

The overall crude worldwide prevalence of ALS is approximately 4.42 per 100,000 (3), and it is estimated that by 2040 the Iranian ALS population will roughly reach 3,000 (4). The first epidemiological study of the Iranian ALS population was published in 2010 reporting on 98 ALS patients evaluated during a 4-year period in outpatient or inpatient departments of Isfahan Medical University located in central Iran (5). Comparing the findings of this study with the worldwide data in a meta-analysis (6) showed that the mean age of onset is lower among Iranian patients (<55 years) compared to Europe and New Zealand (63–65 years) as well as America and East Asia (59 years). In addition, the frequency of bulbar-onset phenotype in the Iranian study (27%) was lower compared to Europe (45% in the Northern areas to 34% in the Western and Southern areas) but closer to the East Asia (28%), Israel (22%), and America (28%) estimates. This finding, along with younger age of onset, might explain the higher survival reported in the Iranian study (48 months) compared to the other areas (25–30 months in Europe and 35 months in North America). Another difference was in the sex ratio (M/F) which was higher in the Iranian study (approximately 2) compared to Europe, North America, and New Zealand (1.22–1.33) but closer to Asia (1.55 in East and 1.72 in West Asia), Uruguay, Libya, and Hawaii (>2). In another study from Mashhad in Northeast Iran, 59 consecutive ALS patients were evaluated (7). A similar age of onset (48 years), sex ratio (1.8), and survival (53 months) were reported, while the bulbar-onset phenotype was even less frequent (15%). The rate of familial ALS was 3.8% in this study which is close to the worldwide estimate (4.7%) (6). Tracheostomy with mechanical ventilation was reported in 20% of patients in this sample. The largest natural history study of Iranian ALS patients, published in 2015, recruited 358 patients from 10 centers in the country (8). Findings were generally in agreement with the previous two reports with the mean age of onset being 52 years and the rate of bulbar-onset and familial ALS at 23% and 3.4%, respectively. The sex ratio was 1.6 which is closer to most other regions. Approximately 23% of patients died during a 12-month follow-up.

### 3.2. Clinical/electrodiagnostic criteria

Diagnosis of ALS is based on clinical judgment supported by the findings from a standard and comprehensive electrodiagnostic study. Such a study should include an evaluation of muscles with different nerve and root innervation in the proximal and distal of at least three limbs, three segments of thoracic paraspinal muscles, and at least one bulbar muscle. El Escorial World Federation of Neurology criteria for the diagnosis of ALS were published in 1994 (9) and revised later in 2000 to improve its sensitivity (10). Awaji criteria (AC) were subsequently proposed in 2008 to improve the integration of electrodiagnostic studies into the existing ALS diagnostic criteria aiming to further increase sensitivity (11). In fact, current evidence indicates that the Awaji criteria are significantly more sensitive than the revised El Escorial criteria (73 vs. 58%, respectively) (12–15).

Despite improved sensitivity, complexity and low inter-rater reliability were still major barriers to the use of the Awaji criteria in a multicenter study (16). To address these limitations, a consensus meeting led by the World Federation of Neurology was held in 2019 to develop a new set of criteria (17). The resulting Gold Coast criteria (Table 1) are believed to be simpler, more sensitive, and more useful in clinical settings than the previous criteria (18–20). Current improvements in the care of ALS patients, especially in the setting of multidisciplinary clinics, necessitate highly sensitive diagnostic criteria that also allow earlier diagnosis. However, it is important to note that, as part of the clinical criteria, ALS mimics and differential diagnoses should be excluded using appropriate evaluations before a diagnosis could be made.

**Table 1:** Gold coast ALS criteria.

**All three must be present:**
1. Progressive motor impairment documented by history or repeated clinical assessment, preceded by normal motor function
2. The presence of at least one of: •Upper^a^ and lower^b^ motor neuron dysfunction noted in the same body region^c^ if only one region is involved •Lower motor neuron dysfunction in at least 2 body regions^c^
3. Exclusion of other better diagnoses

a Upper motor neuron dysfunction is defined by at least one of the following: (1) Increased deep tendon reflexes defined as presence of a reflex in a weak muscle or spread to adjacent muscles. (2) Pathological reflexes (Hoffman sign, Babinski sign, crossed adductor reflex, and snout). (3) Velocity-dependent hypertonicity (spasticity). (4) Slowed, poorly coordinated voluntary movement, not attributable to lower motor neuron weakness or Parkinsonism.

b Lower motor neuron dysfunction is defined by at least one of the following: (1) Clinical muscle weakness and wasting. (2) Electromyography abnormalities that must include both evidence of chronic neurogenic change and evidence of ongoing denervation.

c Body regions (bulbar, cervical, thoracic, and lumbosacral) for lower motor neuron involvement (either clinical or on electromyography) include at least one of the following: (1) Involvement of two limb muscles innervated by different roots and nerves. (2) One bulbar muscle. (3) One thoracic paraspinal muscle.

#### 3.2.1. Recommendations

- The application of the Gold Coast criteria results in an early detection of ALS patients and is recommended for the diagnosis of ALS in clinical practice (grade A).
- The exclusion of ALS mimics is mandatory for the diagnosis of ALS in clinical settings (grade A).

### 3.3. Monitoring of disease progression

#### 3.3.1. Clinical motor scale

The ALS functional rating scale (ALSFRS) and its revised version, ALSFRS-R, are widely used survival predictors and outcome measures in ALS patients (21, 22). ALSFRS-R consists of 12 components on a 0 to 4 scale and has good inter-rater and intrarater reliability (23). Multiple studies have shown that ALSFRS-R can be used as a marker of disease progression with higher progression rates being indicative of lower survival (24–26). However, clinicians should be aware of the limitations of this scale which include relative insensitivity to change in short periods of time (especially under 6 months), the subjective nature of the scale, insensitivity to small changes especially in patients with less severe disease, and lack of unidimensionality which means that the scale should be considered as three separate subscales (bulbar, fine and gross motor, and respiratory) rather than a single scale (27–30).

#### 3.3.2. Motor staging systems

Although multiple staging systems have been developed for the evaluation of various aspects of ALS, the most widely used systems are King's clinical staging and Milano-Torino (MiToS) functional staging systems (31, 32). Both are simple systems that can easily be applied in busy clinical settings. The MiToS system basically divides ALSFRS-R into four domains and counts the number of domains in which the patient has lost his/her independence. These domains include walking and self-care, swallowing, communicating, and breathing. A stage 0 will be applied when functional impairment has not caused loss of independence in any domain. In King's staging, stages 1–3 represent the number of body regions involved, including bulbar, upper limbs, and lower limbs. Stage 4 is when there is nutritional or respiratory failure necessitating gastrostomy or non-invasive ventilation (NIV). Stage 5 in both systems represents death. King's staging can also be derived from ALSFRS-R (33).

#### 3.3.3. Measures of quality of life

The ALS Assessment Questionnaire (ALS-AQ40) is a health-related quality-of-life measure for ALS with high internal reliability and validity (34, 35). A Persian version is also available (36).

#### 3.3.4. Cognitive/behavioral screens

As discussed in the cognitive section below, cognitive/behavioral dysfunction affects more than half of the patients with ALS, leading to frank dementia in approximately 15–20% (37). Cognitive dysfunction in ALS is associated with poor outcomes (38). Several cognitive/behavioral screens are available; however, no single tool has consistently been used in related studies (39–41). The Edinburgh Cognitive and Behavioral ALS Screen (ECAS) is a more comprehensive tool that evaluates a wide range of cognitive functions, including a detailed evaluation of language and social cognition domains. The ALS Cognitive Behavioral Screen (ALS-CBS) is a simpler tool that more specifically measures executive dysfunction (42). Both scales also evaluate behavioral changes based on a caregiver-administered questionnaire. A validated Persian version of the ECAS is currently available (43).

#### 3.3.5. Objective measurement of motor function

The Motor Unit Number Estimation (MUNE) and the Motor Unit Number Index (MUNIX) are quantitative neurophysiological measures that estimate the number of remaining motor units in a muscle. These methods provide a more sensitive method to quantitatively measure ALS progression compared to clinical methods even in pre-symptomatic muscles (44–48). These features make MUNE and MUNIX good biomarkers of disease progression in clinical research; however, clinical scales are more widely available and easy to use in clinical settings.

#### 3.3.6. Recommendations

- Use of ALSFRS-R as a measure of disability is recommended for all ALS patients at baseline and follow-up visits (grade A).
- ALS-CBS or ECAS is recommended for cognitive/behavioral screening of ALS patients at baseline and follow-up visits (grade A).
- Use of disease-specific quality-of-life assessment for ALS such as ALSAQ-40 is recommended in baseline and follow-up visits of all ALS patients in tertiary ALS clinics (grade B).

### 3.4. Differential diagnostic workup

Additional workups in ALS mostly focus on excluding differential diagnoses and constitute an important step in the diagnosis of ALS. Depending on the clinical presentations, various combinations of diagnostic tests might be considered in the evaluation of an individual patient.

#### 3.4.1. Neuraxis neuroimaging

Spinal cord magnetic resonance imaging (MRI) is essential to exclude the most common and important ALS mimics, spinal spondylosis, or other space-occupying lesions. Cervical spondylotic amyotrophy is a relatively rare form of myelopathy that can involve more proximal (C5, C6) or distal (C7, C8, and T1) cervical spinal segments (49). Cervical MRI may reveal T2 hyperintensity in addition to central and foramina canal stenosis in both types (50). On the other hand, comorbid cervical spondylosis and cervical cord compression are more common in ALS patients compared to other neurodegenerative and neuromuscular disorders (51). Spinal MRI also provides diagnostic clues in other ALS mimics, such as radiation myelopathy and Hirayama disease. In multifocal motor neuropathy, another treatable mimic of motor neuron disease (MND), contrast-enhanced MRI of the root and the brachial/lumbosacral plexus increases diagnostic certainty (52).

#### 3.4.2. Serum vitamin B12

A large retrospective chart review study showed that laboratory workup resulted in a change in management in 6% of patients but did not change the diagnosis of ALS in any patient. Assays with higher rates of abnormal findings included complete blood count, vitamin B12, serum creatine kinase, and parathyroid hormone (53).

A few case reports of patients with an ALS-like presentation but with a final diagnosis of B12 deficiency have been reported in the literature (54, 55). However, no cases of vitamin B12 deficiency were reported among ALS mimickers from the Irish and Scottish ALS registries (56, 57). Despite the rarity as an ALS mimicker, considering the treatable nature of the disease and the low cost of screening, it is advisable to test vitamin B12 levels in all patients with a primary diagnosis of ALS.

#### 3.4.3. Parathyroid hormone

There are a few reports of patients presenting with weakness and no or minor systemic symptoms or sensory loss with a primary diagnosis of motor neuron disease/ALS subsequently diagnosed with hypercalcemia and hyperparathyroidism (58–62). In some of these studies, no change in disease course was reported after the removal of the parathyroid adenomas, and death subsequently occurred in 3 years. However, significant improvement in neurologic symptoms after resection of the parathyroid adenoma/hyperplasia was reported in others (58). Thereby, it is reasonable to perform serum calcium, phosphorus, and PTH measurement in all patients with a primary diagnosis of ALS.

#### 3.4.4. Paraneoplastic workup

As a paraneoplastic neurologic disorder, MND is a non-classical presentation (63, 64); thereby, by current definition, definite paraneoplastic MND might only be considered in an MND patient when it is associated with cancer *and* a high-risk antibody. Paraneoplastic MND is very rare, and our knowledge is mostly based on case reports. The most commonly associated antibody is anti-Hu, but occasional reports exist with other antibodies including anti-CV2/CRMP5, anti-Ma2, anti-Yo, and anti-Ri (65, 66). Common underlying malignancies include non-small cell lung cancer, breast cancer, and renal cell carcinoma. There are also reports of associated testicular cancer, ovarian cancer, prostatic carcinoma, and thymoma (65, 67).

Testing for monoclonal gammopathies is controversial in patients with MND. Some studies argue that although monoclonal gammopathies might be more common in ALS patients (68), detecting these gammopathies does not change the management of patients, and immunotherapy or chemotherapy has no effect on the course of MND, hence, no utility in testing for gammopathies (69, 70). On the other hand, other studies suggested that testing might be reasonable since it provides valuable clues for the diagnosis of some common treatable ALS mimics that are associated with gammopathies including motor-predominant multifocal acquired demyelinating sensory and motor neuropathy (MADSAM) and multifocal motor neuropathy. These disorders might be difficult to rule out based on clinical and electrodiagnostic findings (56, 57, 71).

#### 3.4.5. Myasthenia gravis antibodies

There are several reports of myasthenia gravis masquerading as ALS, especially in muscle-specific kinase (MuSK)-associated myasthenia (72–74). Considering the characteristic bulbar involvement with muscle atrophy in MuSK myasthenia, it can mimic bulbar-onset ALS (74).

In three patients with MuSK myasthenia who had a primary diagnosis of ALS, dropped head syndrome or dysphagia was the early symptom. Weakness occurred more acutely and progressed more rapidly in these patients compared to ALS (72).

#### 3.4.6. Lead level

Chronic lead poisoning is another mimicker of motor neuron disease (75, 76). Systemic manifestations, when present, can aid the diagnosis. Neuromuscular manifestations of lead poisoning are varied including polyneuropathy, bibrachial palsy, pure motor neuropathy, and typical motor neuron disease (76).

#### 3.4.7. Hexosaminidase A

Late-onset hexosaminidase A deficiency is an uncommon ALS mimic predominantly presenting with lower motor neuron symptoms (77). A PLS-like presentation has also been reported (78).

#### 3.4.8. Recommendations

- Neuraxis MRI (brain and spinal cord) should be obtained in all patients diagnosed with ALS to exclude common differential diagnoses (grade B).
- Laboratory screening of the common treatable mimics should be performed in patients diagnosed with ALS (considering age, LMN and/or UMN involvement, and bulbar signs in choosing proper tests). These include complete blood cell count (CBC diff), blood urea nitrogen/creatinine, fasting blood sugar, hemoglobin A_1_C, thyroid function tests, liver function tests, calcium, phosphorus, PTH, erythrocyte sedimentation rates, serum B12/folate, serum protein electrophoresis/immunofixation, and serum lead level (grade B).
- In patients with bulbar-onset weakness, anti-acetylcholine and anti-MuSK antibodies might be considered (grade B).
- In selected patients, viral markers including HIV, HTLV1, hepatitis B and C, hexosaminidase A in white blood cells or skin fibroblasts, blood very long chain fatty acids, and paraneoplastic panel might be considered (grade B).

### 3.5. Genetic testing

ALS has a high estimated mean lifetime heritability of approximately 52.3% (79). Familial ALS, i.e., involvement of at least one first- or second-degree relative, comprises approximately 5–10% of all patients (80). Siblings and children of ALS patients are 10 times more likely to develop ALS (81) with their lifetime risk being approximately 1.4% compared to 0.3% in the general population (79). Most cases of monogenic familial ALS are autosomal-dominant. Currently, more than 30 genes have been found to contribute to ALS risk. Mutations in four major genes including SOD1, C9ORF72, TARDBP, and FUS together account for almost 50% of familial ALS cases, mostly in the form of autosomal-dominant inheritance, and 6% of sporadic cases (82). In a recent Italian study, 27% of ALS patients were carriers of an ALS-related variant, affecting 54.8% of familial ALS, and 17.5% of sporadic ALS patients (83). In Iran and many other Asian countries, SOD1 variants are the most prevalent while C9ORF72 variants are the most common in Caucasian population and Northern European countries (82, 84, 85). Alavi et al. in two consecutive studies sequenced SOD1 and C9ORF72 in 60 and 78 ALS cases, respectively. In the first study, SOD1 variants were found in approximately 12% of the cohort accounting for approximately 40% of the familial cases and 4% of the sporadic cases. In the second study, only 2 of the 78 cases (one familial and one sporadic) had a C9ORF72 variant, revealing a frequency of 6% among familial and 2% among sporadic cases.

The underlying genetic variants can also affect the ALS phenotype. In patients with C9orf72 repeat expansion, the bulbar phenotype is more common while the pure upper motor phenotype is rare. More importantly, C9orf72 variants are the strongest determinant of comorbid frontotemporal dementia (FTD) with ALS (up to 88% of cases). Bulbar presentation is less common with SOD1 mutations, but a flail leg phenotype is more frequent in these patients (86). FUS mutations are the most common genetic variants among patients with juvenile ALS (87). Earlier age of onset and a more aggressive course with normal cognition is more common with SOD1-ALS cases as seen in the p.A4V variant, although other variants such as p.D90A may have longer life expectancy (82, 88). Most TARDBP mutations present with a typical ALS phenotype and no cognitive impairment (82).

Considering the high contribution of genetic variants in both familial and sporadic ALS, it is reasonable to offer genetic testing to those ALS patients who have an affected first- or second-degree relative. On the other hand, uncertainties and complexities in the interpretation of genetic findings, including incomplete penetrance, multiple genetic variants in an individual, poor genotype–phenotype correlation, and pleiotropy, raise important ethical considerations that restrict genetic testing for sporadic cases (89). Nevertheless, genetic testing could be discussed upon the patient's request. One benefit of genetic testing is that it allows patients to be included in related therapeutic clinical trials such as SOD1-ALS (90). In addition, genetic testing is the main diagnostic tool to differentiate some ALS mimics, especially spinobulbar muscular atrophy or Kennedy's Disease, which presents in male patients with slowly progressive pure lower motor neuron syndrome and bulbar involvement. Diagnosis is established by the demonstration of CAG trinucleotide repeat expansion in the androgen receptor gene (91).

#### 3.5.1. Recommendations

- Since genetic factors affect the age of onset, progression rate, survival, and disease phenotype of ALS patients, genetic testing should be offered to ALS patients with an affected first- or second-degree relative (grade C).
- For patients with no family history of ALS, genetic testing should not routinely be offered; however, it could be discussed upon patient request (grade B).
- We strongly recommend against genetic testing in asymptomatic individuals (grade A).

### 3.6. Disease-modifying treatments

#### 3.6.1. Pharmacological agents

Riluzole, a benzothiazole derivative, is the first disease-modifying drug approved by the US Food and Drug Administration (FDA) in 1995 for the treatment of ALS. Multiple clinical trials and systematic reviews showed that riluzole increases overall survival by 3 months and improves bulbar and limb function; however, no significant improvement was found in muscle strength (92). Some studies suggested that increased survival is significantly higher in bulbar-onset compared to limb onset phenotypes (92). The effect of riluzole on the quality of life of ALS patients has not specifically been studied. Riluzole is less beneficial in patients of 75 years or older, those with more severe respiratory involvement (forced vital capacity (FVC) < 60%), or longer disease duration (>5 years of onset), although it is well tolerated in these patients (93, 94). Adverse effects of riluzole include nausea, asthenia, vomiting, diarrhea, anorexia, dizziness, and low hemoglobin (92).

Edaravone, the second drug approved by the US FDA in 2017 for ALS disease modification, is a pyrazolone, with free radical scavenger and neuroprotective properties. The effectiveness of edaravone was shown in a *post-hoc* analysis (95) performed on the data from the original study (96) and later confirmed in an independent prospective study (97). Therefore, the use of edaravone has been limited to this specific subgroup of patients with (a) age between 20 and 75 years, (b) living independently (grade 1 or 2 in the Japan ALS severity scale), (c) 1–4 scores decrease in ALSFRS-R in the last 12 weeks, (d) score 2 or higher in each item of ALSFRS-R, (e) FVC ≥ 80%, (f) disease duration ≤ 2 years, and (g) probable or definite ALS diagnosis, in addition to absence of any dyspnea, orthopnea, spinal surgery, or renal insufficiency (97). The results from a recent systematic review of three randomized studies on edaravone were indicative of slower disease progression, with 1.63 points on the ALSFRS-R score in edaravone compared to placebo (98). Two recent retrospective large real-world studies showed conflicting results. In the first multicenter study in Germany, the authors reported no difference in disease progression, time to ventilation, or survival between patients receiving edaravone plus riluzole compared to those treated only with riluzole (99). The second study in the US compared the overall survival of ALS patients treated with edaravone to those not treated with edaravone and found a 27% survival benefit (29.5 months vs. 23.5 months) in those treated with edaravone (100).

Edaravone is administered as an intravascular infusion of two 30 mg vials per day for 14 days followed by a drug-free interval of 14 days in the first treatment cycle which is reduced to 10 days in subsequent cycles. An oral suspension has recently been approved by the US FDA. Adverse effects of edaravone are minimal and include headache, bruising, gait disturbance, eczema, respiratory disorder, and glycosuria (101).

On September 2022, the US FDA approved Relyvrio, a combination of sodium phenylbutyrate and taurursodiol for ALS disease modification. It is believed that it causes a reduction in neuronal death via effects on mitochondria and endoplasmic reticulum. In the first phase 2 clinical trial, a slowing of functional decline was observed based on the ALSFR-R score (1.24 per month in the treatment vs. 1.66 in the placebo group) although there was no significant improvement in any of the secondary outcomes including muscle strength, slow vital capacity, hospitalization rate or time to death or tracheostomy (102). ALS patients with less than 18 months of symptom onset under simultaneous treatment with riluzole, edaravone, both, or none were recruited in this study. The treatment regimen consisted of 3g of sodium phenylbutyrate and 1g of taurursodiol once daily for 3 weeks followed by a twice-daily dosing for 21 weeks. The following open-label extension study which assigned all patients to treatment for 35 months showed that the tracheostomy/permanent-assisted ventilation-free survival was significantly longer in patients primarily randomized to the treatment group (25.0 months in the treatment-first vs. 18.5 months in the placebo-first group) (103, 104). Therefore, an early start of Relyvrio increased survival for about 6.5 months.

Following the successful development and US FDA approval of nusinersen, an antisense oligonucleotide for the treatment of spinal muscular atrophy (105), tofersen, an antisense oligonucleotide targeting SOD1gene product, was developed by Ionis/Biogen and tested in a phase III trial on 108 participants with SOD1-ALS (106). Although clinical improvement was not evident after 28 weeks, 12-month data from the open-label extension study showed a slowing in the rate of clinical motor and respiratory decline (107). In addition, neurofilament light, as a marker of disease progression, decreased significantly in cerebrospinal fluid. These findings led to accelerated approval by the US FDA in April 2023. Once available, this drug could be of special interest to Iranian and other Asian ALS patients due to the higher rate of SOD1 mutations in these populations.

##### 3.6.1.1. Recommendations

- Treatment with riluzole is strongly recommended for all ALS patients regardless of their disease status (grade A).
- Edaravone should be considered in selected independent ALS patients with 2 years or less of disease duration, mild severity based on ALSFRS-R, and no significant respiratory symptoms (FVC ≥ 80%) (grade A).
- Sodium phenylbutyrate/taurursodiol as a single or add-on therapy is recommended for all ALS patients with less than 18 months of symptom onset (grade B).
- Tofersen as a single or add-on therapy is recommended for all familial SOD1-ALS patients (grade C).

#### 3.6.2. Unapproved treatments

Stem cell therapy is expected to be used for multiple purposes in ALS including preventing further neurodegeneration by producing growth factors, clearance of toxic materials, providing immunomodulatory support, and potential replacement of damaged motor neurons (108). Despite promising results in preclinical studies (109–115), clinical studies are yet inconclusive considering their poor design and small sample size. A recent systematic review and meta-analysis of clinical stem cell studies in ALS found only a transient improvement in ALSFRS-R in studies using intrathecal mesenchymal stromal cells (116, 117). Unexpectedly, this improvement was accompanied by a respiratory worsening based on FVC measurement in these studies. Stem cell therapy in any form has not shown any significant effect on the survival or quality of life of the participants. The major source of bias in these studies was unblinded outcome assessment. Intra-spinal transplantation of neural stem cells has shown a transient improvement in one and no significant effect on disease progression in another study (117, 118). Intrathecal injection of neurotrophic factor-secreting mesenchymal stem cells which showed promising results in a phase 2 study failed to show a significant effect on primary endpoints in the recent phase 3 clinical trial that included 196 ALS patients (119).

##### 3.6.2.1. Recommendations

- Stem cell therapy in any form is not recommended in clinical settings (grade C).
- Considering the inconsistent and contradictory results of clinical studies, we also recommend against the use of stem cells in clinical research settings at this time. Further preclinical studies to better characterize the preferred cell type and specifications, dosage, and administration route are needed (grade C).

### 3.7. Multidisciplinary care management

A multidisciplinary approach is currently considered the standard of care in the management of ALS, which allows providing high-quality care and improving survival and quality of life, while also decreasing complicated hospitalizations and healthcare costs (120–122). However, most patients do not receive timely care in a multidisciplinary ALS clinic. A study in Ireland showed that the time interval between ALS diagnosis to the first visit to a multidisciplinary clinic is approximately 19 months (123). Patients with ALS have several health needs that could best be managed in a multidisciplinary clinic including non-invasive ventilation (NIV), sialorrhea, secretions management, percutaneous endoscopic gastrostomy (PEG) feeding, and behavioral and cognitive disturbances.

While curative treatments are not available for ALS, care in multidisciplinary clinics can increase the survival and quality of life of patients (124). Behavioral and cognitive impairment are important conditions that affect many ALS patients and can disrupt care management in a non-multidisciplinary care setting leading to decreased survival. However, these symptoms can be recognized at earlier stages in a multidisciplinary clinic setting and managed accordingly to decrease their detrimental impact (125). Another important advantage of multidisciplinary ALS care is the initial assessment by a nutrition specialist and the provision of personalized nutritional support that can decrease the rate of severe malnutrition in these patients (126). Rehabilitation is an integral component of a multidisciplinary ALS clinic that can assist patients to continue their independent function safely, enabling them to perform to their fullest potential despite ALS. It is also useful to include a palliative care specialist to manage pain and end-of-life care (127). Social workers have been an important part of the multidisciplinary ALS team in many countries; however, this service is not widely available or covered by insurance in Iran. Further local studies are needed to evaluate the acceptance and role of social workers in the management of ALS patients.

In special situations, multidisciplinary care could be provided via telehealth measures; however, further local studies are needed to evaluate its efficiency, feasibility, and acceptance by patients and providers (128).

#### 3.7.1. Recommendations

- All patients must be referred to a multidisciplinary clinic (tertiary center) as soon as an MND diagnosis is made (grade B).
- Multidisciplinary ALS clinics should include neurologists, pulmonologists, speech and language therapists, physical and rehabilitation medicine specialists, nutritionists, gastroenterologists, psychiatrists, and psychologists (grade B).
- All ALS patients should have access to multidisciplinary care. In areas with limited availability of multidisciplinary ALS clinics, a team of local neurologists, pulmonologists, nutritionists, rehabilitation specialists, and psychiatrists must form a local network to manage ALS patients (grade B).

### 3.8. Rehabilitation

The aim of rehabilitation in ALS patients is to improve function, symptoms, quality of life, and survival. Designing a rehabilitation protocol is difficult in patients with ALS because their functional status changes rapidly. Six stages of progression are traditionally being considered in these patients (129). Recommended exercise and rehabilitation measures for each stage are shown in Table 2.

**Table 2:** Rehabilitative measures based on Sinaki stages.

**Stage**	**Weakness**	**Functional impairment**	**Rehabilitative measures**
1	Mild	Fully independent	•Stretching exercise and active range of motion of the affected joints •Strengthening of unaffected muscles •Aerobic activities
2	Moderate	Severe only in some areas	•Stretching exercise and active range of motion of the affected joints •Strengthening of unaffected muscles •Aerobic activities •Appropriate equipment and assistive devices
3	Severe in some muscle groups	Ambulatory	•Ambulation aids such as walker or manual wheelchair
4	Severe	non-ambulatory but independent	•Passive range of motion and active assisted range of motion exercises •Strengthening exercises and active range of motion of any unaffected muscle •Pressure relief surfaces
5	Severe, more widespread	Dependent	•Transfer assistance •Family education •Wheelchair when out of bed •Pain management •Semirigid collar
6	Severe, generalized	Completely immobile	•Hospital bed •Skilled caregivers •Palliative care

The most widely used rehabilitation modality in ALS is exercise. Exercise has traditionally been avoided in ALS patients believing it might worsen the course of the illness. More recent studies including recent systematic reviews and meta-analyses have shown that range of motion and stretching exercise is safe and effective in these patients which led to the inclusion of exercise as part of standard ALS care (130–132). Exercise may help to maintain function and prevent contracture and pain and, in the long term, can improve functional ability and pulmonary capacity. Endurance/aerobic exercise seems to be superior to resistance exercise in ALS, and moderate-intensity exercise is preferred over high-intensity exercise (131). Patients should be encouraged to start daily exercise early in the disease course. The intensity of exercise should be modified such that to avoid fatigue, dyspnea, or cramp which are signs of overuse weakness. Pain and fatigue that continue longer than 30 min after exercise are other indicators that show the exercise program must be modified (133). On the other hand, prolonged interruption of rehabilitation may accelerate functional motor decline in ALS patients (134).

Assistive devices and orthoses such as ankle foot orthosis, night splint, wrist extension orthosis, thumb positioning orthosis, and cervical collar, as well as adaptive equipment and assistive devices including cane, walker, and wheelchair, are used to assist in the improvement of function and mobility in ALS patients.

Evidence is low or lacking about other modalities such as breathing exercises, resistance exercises, aqua therapy, electrical stimulation, and ultrasound (131).

#### 3.8.1. Effect of rehabilitation on pain, spasticity, and fatigue

Contracture and immobilization are important contributors to pain among ALS patients. Therefore, night-splints, shoulder approximation sleeves, and dynamic splints have been proposed to help keep ankles and hands in a neutral position, prevent shoulder subluxation, and reduce the risk of contractures. Regular skin check is necessary to prevent complications including pressure sores or pain. Proper lumbar support for wheelchair-bound patients may reduce low back pain. Gait training, appropriate transfer techniques, and modification of home and workplace to reduce the risk of falls and injuries may help in maintaining function and preventing pain (135–138). Physical modalities including ice and heat may be used to reduce pain.

In the management of spasticity, rehabilitation measures are the most important options. Stretching and a range of motion exercises, cold pack, transcutaneous electrical nerve stimulation, shock wave and ultrasound, night-time neutral position splinting, and hydrotherapy are suggested; however, more studies are needed (139, 140).

Exercise, work simplification, and energy conservation techniques including pacing and regular rest periods between activities may be of assistance in reducing fatigue in ALS patients. Using proper orthosis with light weight material and wheeled walkers instead of standard walkers is also recommended. When appropriate, manual or powered wheelchairs or scooters can be used to reduce fatigue. A custom-fitted wheelchair is a better choice to support the spine and provide pressure relief.

#### 3.8.2. Recommendations

- Range of motion and stretching exercises are effective and recommended in all ALS patients (grade B).
- Ambulatory patients might benefit from aerobic and endurance exercise (grade A).
- Moderate-intensity strengthening exercises should be reserved for patients with high functional status (grade A).
- Exercise should not cause pain or fatigue that lasts more than 30 min, overuse weakness, cramps, or dyspnea (grade E).
- Proper use of splints and sleeves reduces the risk of painful contracture and subluxation (grade E).
- Gait training programs and home and workplace modifications are recommended to prevent falls and injuries (grade E).
- Patients' and caregivers' education should include appropriate transfer techniques and proper wheelchair back support (grade E).
- Use of orthoses and assistive and adaptive devices should be considered on an individual basis (grade E).
- Range of motion exercise and stretching as well as proper orthosis or physical modalities are useful to decrease spasticity (grade E).
- Interventions to reduce fatigue include energy conservation techniques, change to lightweight braces, proper assistive devices and mobility aids, and exercise programs including stretching, endurance, and aerobic exercise (grade E).

### 3.9. Management of swallowing, communication, and nutrition

#### 3.9.1. Speech therapy evaluations and interventions

Speech motor problems, although more severe and frequent in bulbar-onset form, have a lifelong prevalence of over 80% in all ALS patients which lead to complete loss of oral communication skills in 75–95% of patients. All subsystems are involved in ALS, especially respiration, phonation, articulation, and resonance. Bulbar weakness leads to anarthria after about 18 months (141). Cognitive dysfunction, especially involving executive, social, and language domains, also accounts for loss of communication abilities in ALS patients.

Dysarthria in ALS is typically of mixed type with varying combinations of flaccid and spastic features, usually starting with nasality leading to a decrease in speech rate and loss of intelligibility in later stages. More severe bulbar weakness might lead to hypernasality and a change in voice quality; nevertheless, phonation is preserved even in later-stage ALS (142).

High-quality studies are lacking about the management of dysarthria and dysphagia in ALS despite their universal presence in ALS patients. In the early stages, training patients to speak more slowly could be helpful to increase intelligibility and adapt to respiratory weakness. Strengthening exercise of the bulbar muscles and diaphragm is controversial; however, the general exercise rules stated in previous sections could reasonably be applied here (143–145). Palatal lift has been suggested for patients with significant dysarthria due to hypernasality. In advanced stages, many patients with ALS will need to use augmentative assistive communication technologies. There is a notably high acceptance rate of these technologies among ALS patients and reportedly 46% of patients continue to use the technology during their last week of life (13–15).

Dysphagia, mostly of oropharyngeal type, is an early presentation in 20–30% of the patients. In later stages, nearly all are affected which leads to decreased swallowing safety and efficacy (146). Patients with mild-to-moderate dysphagia might benefit from swallowing aid techniques. Recently, the “Ishizaki Press Method” has been shown to be effective for dysphagia in a patient with severe ALS (147). This method involves the application of finger pressure on specific maxillofacial points and is believed to play a role in triggering the swallowing reflex.

##### 3.9.1.1. Recommendations

- It is recommended that all patients with ALS be evaluated by an expert speech and language pathologist at the first signs of disease and treated and followed up every 3 months or as indicated in speech therapy service during the mild-to-moderate stages (grade B).
- Dysphagia intervention should be considered according to the disease stage:

a. In the early stages of ALS with normal swallowing, speech pathologist consultation should be offered (grade B).
b. In the presence of occasional problems with eating and drinking, interventions include modification strategies, postural adjustments, and swallowing maneuvers (grade B).
c. When encountering moderate problems with eating and drinking, dietary consistency changes are recommended (grade A).
d. In the more severe stage, feeding tube placement will be a safe option. High-viscosity liquids should be handled with caution (grade B).
e. For all stages, with or without eating ability, oral hygiene should be practiced throughout the day (grade A).

- For dysarthria, using slow speech rate, exaggeration of articulation, improvement of respiratory efficiency through phrasing, tongue strengthening exercises, and diaphragmatic exercises are recommended (grade B).
- At any stage when a patient with ALS cannot communicate the use of augmentative assistive communication strategies should be considered to enhance the quality of life (grade B).

#### 3.9.2. Sialorrhea

Drooling or sialorrhea is not only a troubling symptom in patients with ALS but also increases the risk of aspiration pneumonia. The prevalence of sialorrhea in ALS patients is approximately 20–50% (148). Normal daily production of saliva is about 1.5 liters, and its clearance depends on the function and strength of the bulbar, facial, and buccal muscles. Dysphagia and weakness of these muscles due to ALS lead to the pooling of saliva and eventually drooling.

Excess salivation directly or indirectly influences ALSFRS-R scores, mostly in the first three items (speech, salivation, and swallowing); therefore, the overall function and quality of life will improve with the successful management of sialorrhea (149). Moreover, in ALS patients who are using NIV, the pooling of oral secretions may lead to decreased tolerance and efficiency of NIV, the consequences of which are hypoxia and aspiration pneumonia. It has been shown that patients with normal oral secretion scores better tolerate NIV and have longer survival compared to those with severe sialorrhea (150). Physicians should be aware that although sialorrhea is troublesome, care should be taken to avoid overtreatment leading to dry mouth since it reduces dental hygiene and aggravates swallowing difficulties.

Different options for the management of sialorrhea include behavioral techniques (e.g., deliberate swallowing), frequent suction, anticholinergic agents, botulinum toxin injection, and radiotherapy. Anticholinergic agents are the most common first-line treatment used in the management of sialorrhea. In one study, 61% of patients with sialorrhea showed some degree of improvement (151). However, side effects especially in elderly patients remain a major concern (152).

The second therapeutic option for the management of sialorrhea resistant to anticholinergics or when side effects develop is local botulinum neurotoxin injection (153). Its efficacy has been approved in a number of studies with effects lasting up to 4 months and side effects being negligible (154). There is generally no significant difference between botulinum toxins type A and B (155).

Radiation to the salivary glands is another therapeutic option for the management of sialorrhea that should be reserved for patients who are refractory to other options (136, 156). The total dose of 20Gy divided over 4–5 fractions seems to be the most effective; however, tolerability might be an issue in some patients in whom lower doses could be tried (e.g., 8 Gy in one fraction) (120, 157).

##### 3.9.2.1. Recommendations

- Sialorrhea should be actively evaluated and appropriately managed in ALS patients to improve quality of life and survival (grade A).
- In the management of sialorrhea, it is important to avoid dry mouth which may lead to poor dental/oral health and dysphagia (grade A).
- Anticholinergic agents are recommended as the first-line therapeutic option (grade A).
- In cases of anticholinergic resistance or significant side effects, botulinum neurotoxin injection into the salivary glands should be considered (grade B).
- Salivary gland radiation should be reserved for patients who are refractory to anticholinergic agents and botulinum neurotoxin injection (grade B).

#### 3.9.3. Nutrition interventions

Nutritional status has an important role in determining the prognosis among ALS patients (158), but unfortunately, malnutrition affects 16–55% of ALS patients (159) which together with progressive muscle wasting leads to significant weight loss. Therefore, regular nutritional assessment at diagnosis and every 3 months is recommended by all available guidelines (120, 136, 153). The assessment includes a history of recent weight loss, current food intake, and dysphagia assessment as well as lipid profile, nutrient levels, and evaluation of nutrition-related complications. Although weight and body mass index (BMI) measurements are helpful in detecting malnutrition, body composition analysis using dual-energy X-ray absorptiometry (DEXA) or bioelectrical impedance analysis provides valuable information for distinguishing between loss of fat tissue versus muscle atrophy. More frequent monitoring might be necessary in patients with known malnutrition or dysphagia (160). Any indication of weight loss or malnutrition should be treated aggressively as soon as diagnosed considering the grave consequences. Food fortification is usually the first step due to its convenience and fewer complications (160). Measurement of energy expenditure in ALS patients can inform the decision to offer various nutritional interventions. Indirect calorimetry provides a more reliable measurement of energy expenditure; however, if not available, this could be estimated via the Harris–Benedict equation using the patient's nutritional status, malnutrition history, physical activity, and ventilation condition (161). Generally, non-ventilated patients have higher energy requirements.

A water swallow test or volume viscosity swallow test could be used for nutritional evaluation of dysphagia. In ALS patients, dysphagia is usually more prominent with thin liquids; therefore, food with soft, semisolid, or semiliquid consistency is preferred (153, 160, 162). In moderate dysphagia, dietary counseling may include texture modification as well as patient education to perform postural maneuvers, chin tuck, head rotation, and throat clearing (160, 163). Home parenteral nutrition is generally not indicated in ALS patients (153, 160, 164).

##### 3.9.3.1. Recommendations

- Regular nutritional assessment by a nutritionist including nutritional history, clinical examination with swallowing evaluation, and weight and BMI measurement should be performed at baseline and at least every 3 months in all patients (grade A).
- Objective measurement of body composition and energy expenditure might be considered on an individual basis (grade A).
- Nutritional counseling includes a discussion of food fortification, oral nutritional supplementation, and the possible need for early enteral nutrition such as PEG. Food enrichment is recommended when weight loss, fatigue, or effortful feeding presents, while oral nutritional supplementation is recommended for patients with unmet energy and nutrient requirements (grade A).

#### 3.9.4. Gastrostomy feeding

Considering the high frequency of swallowing impairment in ALS, evaluation of swallowing function should be performed early in the disease course in all patients. Patients with signs of swallowing impairment will benefit from a more comprehensive instrumental swallowing evaluation, including a video fluoroscopic swallow study (VFSS) or fiberoptic endoscopic evaluation of swallowing (FEES). During clinic visits, assessment of swallowing function may include eating and swallowing questionnaires, dietary intake, examination of bulbar function and dysphagia, pulmonary function and airway clearance, and estimation of aspiration risk (165). Swallowing function could also be estimated based on the ALSFRS-R, pulmonary function tests, and regular body weight measurements (166, 167). Eating assessment tool-10 (EAT-10) is a validated measure to detect unsafe swallowing. Patients with EAT-10 scores of 8 or higher are at three times greater risk of aspiration (168). Previous guidelines introduced weight loss of at least 10% relative to baseline as an indication for PEG placement in ALS patients (136). Recent large prospective studies confirmed this indication and showed that patients with weight loss of <10% had better survival after PEG placement (169). Similarly, a BMI of <18.5 is a marker of undernutrition and an important nutritional survival marker for ALS which serves as a guide for nutritional interventions (170). Another important consideration in the decision for PEG placement is the respiratory function status. Decreased FVC of 50% or less almost precludes PEG placement because of the intolerance to the required sedation at this stage. A large retrospective study combined with a meta-analysis of the results of all previous studies showed that a significant increase in survival follows PEG placement, especially when FVC is 50% or more than predicted at the time of the procedure (171). Another meta-analysis compared the efficacy and safety of PEG vs. nasogastric tube (NGT) in patients with various causes of swallowing impairment and found that intervention failure occurred less with PEG compared to NGT; however, there was no significant difference between the groups regarding rates of mortality, adverse events, weight change, pain, or ease of learning (172). Expectedly, compared to NGT, PEG was more convenient for patients with less interference with social activities. Therefore, considering the clear benefits, NGT could be considered if PEG is not practicable.

##### 3.9.4.1. Recommendations

- Enteral tube feeding should be considered if there is a failure of management by speech therapy or diet modification measures with weight loss approaching 10% of baseline, BMI < 18.5, unsafe swallowing in instrumental tests, or if FVC is approaching 50% irrespective of the degree of swallowing impairment (grade A).
- PEG and NGT have similar effectiveness in maintaining good dietary intake, but regarding quality-of-life measures, PEG is superior (grade A).

### 3.10. Assessment and management of respiratory failure

An updated Cochrane review showed that NIV improves the quality of life and prolongs survival of ALS patients with a normal to moderately impaired bulbar function by 205 days, although it did not improve survival in those with severe bulbar weakness (173). Therefore, it is important to determine the need and timing of NIV in ALS patients. After the initial ALS diagnosis, the indicators of respiratory failure should carefully be examined, although many patients might not manifest respiratory symptoms. FVC measurement is traditionally considered the gold standard of respiratory assessment; however, due to the high frequency of bulbar and lip weakness in ALS patients, a standard spirometry cannot be performed in some patients or might generate inaccurate information. In fact, some studies propose that other tests such as slow vital capacity (SVC), supine FVC, maximal inspiratory (MIP) and expiratory pressures (MEP), peak cough flow (PCF), and sniff nasal-inspiratory pressure (SNIP) are more accurate in these patients for the evaluation of respiratory function and decision about NIV initiation (174–177). Maximum cough expiratory flow demonstrates the effectiveness of cough and airway clearance. PCF can be considered as an indicator of expiratory muscle function which measures how voluntary cough can affect the risk of aspiration pneumonia.

Some ALS patients may have normal FVC measurements despite nocturnal desaturation. Nocturnal desaturation indicates weakness of the respiratory muscles and can be considered an indication for the use of NIV. Therefore, more frequent nocturnal oximetry is advisable in the respiratory assessment of ALS patients. ALS patients usually have a progressive decline in sleep quality which affects the quality of life and survival of these patients. Considering the limitations of performing a standard polysomnography in ALS patients, sleep apnea could be assessed by a combination of oximetry or home respiratory polygraphy plus transcutaneous or end-tidal carbon dioxide monitoring (178, 179).

The first evaluation of respiratory function usually consists of clinical history and examination aiming to reveal any tachypnea, dyspnea, orthopnea, paradoxical respiration or use of accessory muscles, nocturnal desaturation, and daytime headache or sleepiness. The Epworth sleepiness scale could be used for the evaluation of daytime sleepiness (scores above 9). Any sign of respiration impairment should then trigger further evaluation using one of the abovementioned modalities. Usually in the presence of one of the findings such as FVC < 80%, SNIP < 40 cmH_2_O, pCO_2_ > 45 mmHg, or nocturnal desaturation, the use of NIV is on the agenda (136). FVC decreases more slowly in patients who start NIV earlier (180). In fact, more recent studies have shown that even earlier initiation of NIV, with FVC ≥ 80%, further increases survival in ALS patients (181, 182). Factors such as the use of cough aids, control of secretions, and proper nutrition can improve the effectiveness of NIV.

Respiratory infections are frequent in ALS patients due to inactivity, sialorrhea, cough inefficiency, and dysphagia. In such cases, the risk of developing pneumonia can be minimized by methods such as vaccination against influenza and pneumococcus. Cough is the main airway protection mechanism, but it is considerably weak in many ALS patients. Assisted cough devices are useful when peak cough flow falls below 270–300 L/min (183). Existing evidence confirms the beneficial role of these physiotherapy interventions in improving respiratory complications and increasing the survival of ALS patients (184). Studies show that mechanical insufflation/exsufflation and the breath-stacking technique are associated with a reduction in adverse respiratory complications (185, 186).

#### 3.10.1. Recommendations

- Signs of respiratory impairment such as daytime headache, sleep disturbances, weak cough, and labored breathing should be examined in all ALS patients on every visit and at least one or two times every 3 months (grade A).
- FVC monitoring, SNIP, maximum inspiratory/expiratory pressure, or supine FVC testing (in patients with normal upright respiratory tests) must be done in all patients at least once every 3 months (grade A).
- NIV should be initiated in patients with FVC < 80%, SNIP < 40 cmH2O, pCO2 > 45mmHg, significant desaturation on overnight oximetry, or when signs/symptoms of respiratory weakness are present (grade A).
- Patients and caregivers should receive education about breath-stacking techniques.
- Patients with decreased cough or PCF of less than 270 L/min in whom medical treatment has failed to manage secretions or is not an option, assisted cough devices should be offered (grade B).

### 3.11. Symptom management

#### 3.11.1. Pain

Pain is extremely common during the course of ALS which sometimes might start even before the onset of motor dysfunction (187). Depression and reduced quality of life have a significant association with pain in ALS patients. The first step in pain management is proactive screening which means that the patients' self-report is insufficient, and physicians should actively inquire about pain (153, 188). The second step is to identify the source. Nociceptive pain due to secondary causes (e.g., joint deformities and skin pressure) is the most common type of pain especially as disability increases. Nociceptive pain responds well to non-steroidal anti-inflammatory drugs and paracetamol which make the first line of treatment in these patients (189–191). Nociceptive joint pain can also be treated with local injection of steroids or lidocaine (188, 192). Gabapentin, pregabalin, and tricyclic antidepressants are recommended when pain has a primarily neuropathic nature (120, 188, 193). In a more advanced stage of the disease, opioids are reported to be effective (190, 191). Cramps and spasticity are other major causes of primary pain in ALS and should be managed accordingly (188, 194).

##### 3.11.1.1. Recommendations

- In the treatment of nociceptive pain, non-steroidal anti-inflammatory drugs and paracetamol should be considered in the first line. As the second line, intra-articular steroids or lidocaine injection might be considered (grade B).
- Gabapentin, pregabalin, and tricyclic antidepressants could be considered in patients with a neuropathic type of pain (grade B).
- In selected patients with advanced disease, administration of morphine in an inpatient or palliative care setting reduces pain and improves the patient's quality of life (grade B).

#### 3.11.2. Spasticity, cramp, and fasciculation

Spasticity limits patients' ambulation, disturbs physical therapy, and causes pain. Baclofen, followed by tizanidine and benzodiazepines is the most common medicine used to treat spasticity (120). Intrathecal baclofen may only be considered in intractable cases, although evidence is insufficient (136). Focal botulinum neurotoxin injection in smaller doses might be helpful in selected patients (195). Tetrahydrocannabinol or THC-containing cannabinoids have been approved for the management of spasticity in other neurologic diseases, and there are reports of their effectiveness in ALS patients as well (196). Levetiracetam (1500–3000 mg/day) is well tolerated and showed positive effects on reducing spasticity due to ALS in an open-label study (197).

Cramp is another common cause of pain, sleep disturbances, and fatigue in ALS patients (187). The effectiveness of low-dose mexiletine (300 mg/day) in reducing cramps has been shown in well-designed studies and a recent systematic review (198). Electrocardiography and liver enzyme monitoring are necessary for patients using mexiletine. Quinine sulfate (200–500 mg/day) might be used in ALS patients based on the available evidence of its effectiveness on cramps in general; however, numerous serious side effects, such as bradycardia, cardiac arrhythmias, prolongation of the QT interval and thrombocytopenia, and drug interactions, limit its use (199). It is uncertain if baclofen and gabapentin can improve cramps (190), although they are commonly used in clinical practice. Levetiracetam (1500–3000 mg/day) decreased muscle cramps in an open-label study (197). Magnesium supplement is not effective and not recommended in patients with ALS due to the possible side effects (200).

Fasciculations are not usually bothersome in ALS patients and do not require medical intervention. There are no approved medications to treat fasciculation; however, considering the acceptable side effect profile, gabapentin could be used if fasciculations are bothersome.

##### 3.11.2.1. Recommendations

- Baclofen, tizanidine, benzodiazepines, THC cannabinoids, and levetiracetam can be considered pharmacological treatments for spasticity (grade B).
- A low dose of mexiletine (300 mg/day) is effective in the treatment of cramps in ALS patients (grade B).
- Levetiracetam (1500–3000 mg/day) can be used for treating spasticity and cramps with a favorable safety and tolerability profile (grade B).
- A low dose of quinine sulfate (200–500 mg/day) is effective for the treatment of cramps and could be considered a last resort, considering its serious side effects (grade B).

#### 3.11.3. Fatigue

Fatigue due to multiple causes is a common symptom of ALS. Respiratory insufficiency is an important cause that should be addressed properly, but respiratory exercise does not improve fatigue in these patients. Depression, sleep disorders, and spasticity are other main causes of fatigue; however, there is no evidence that certain antidepressants or hypnotics could improve fatigue. Riluzole treatment has also been associated with fatigue as one of its side effects. The possible role of riluzole in fatigue with respect to its modest effects on survival should be discussed with patients who are experiencing significant fatigue; thereby, the reduction or discontinuation of the medicine may be considered (92). Modafinil (up to 300 mg/day) has been shown to be effective for fatigue with mild tolerable side effects such as headache, nervousness, nausea, and insomnia (201). There is no evidence regarding the role of diet modification such as a high-calorie diet in the treatment of fatigue in patients with ALS; however, creatine supplementation might reduce its severity (202).

##### 3.11.3.1. Recommendations

- The causes of fatigue such as respiratory weakness, sleep disturbances, spasticity, and depression should be addressed properly (grade B).
- When other measures fail, significant fatigue in patients receiving riluzole could be managed by reducing the dose or discontinuation of riluzole. Benefits versus modest survival benefits should be discussed with the patient (grade B).
- Modafinil (up to 300 mg/day) is probably effective in the treatment of fatigue with an acceptable tolerability profile (grade B).

#### 3.11.4. Pseudobulbar affect

Pseudobulbar affect is seen in approximately one-third of ALS patients and may lead to social isolation in some patients (203). Antidepressants including citalopram, sertraline, fluoxetine, paroxetine, amitriptyline, nortriptyline, imipramine, fluvoxamine, venlafaxine, duloxetine (60 mg daily), and mirtazapine are commonly used in the treatment of pseudobulbar affect of any cause (204). A combination of dextromethorphan and quinidine (20 mg/10 mg, every 12 h) has been approved by the US FDA for the treatment of pseudobulbar affect, and its effectiveness in ALS patients was shown in relatively large clinical trials (205). Generally, this combination is well tolerated in studies without significant cardiac or respiratory adverse events. The most common adverse effects include nausea, diarrhea, dry mouth, headache, dizziness, somnolence, and fatigue (206).

##### 3.11.4.1. Recommendations

- Dextromethorphan plus quinidine 20 mg/10 mg is effective for the treatment of pseudobulbar affect in ALS patients; however, it is not commercially available in Iran (grade B).
- Antidepressants such as amitriptyline, nortriptyline, fluvoxamine, citalopram, mirtazapine, venlafaxine, duloxetine, and dextromethorphan/bupropion could be considered for the treatment of pseudobulbar affect in ALS patients (grade B).

#### 3.11.5. Sleep disorder

Sleep quality is frequently impaired in ALS patients with the main causes being immobilization, muscle cramps, fasciculations, depression, anxiety, respiratory muscle weakness, and nocturnal hypoventilation. Treating specific underlying causes of sleep disturbances is essential in ALS patients. Antidepressants especially mirtazapine (15mg at bedtime) improve sleep quality and reduce anxiety and depression. In patients with sialorrhea, tricyclic antidepressants such as amitriptyline are preferred because of their beneficial effect on sialorrhea. Zolpidem (10mg at bedtime) is the preferred benzodiazepine in ALS patients considering its low risk of respiratory depression (207). In patients with respiratory weakness, NIV significantly improves sleep quality and decreases daytime sleepiness, thereby enhancing quality of life (208).

##### 3.11.5.1. Recommendations

- Antidepressants such as mirtazapine (15 mg at bedtime), tricyclic antidepressants (particularly in patients with sialorrhea), and benzodiazepines (preferably zolpidem) can improve insomnia in ALS patients (grade B).
- Respiratory evaluation is necessary before starting any sedatives, especially benzodiazepine, for ALS patients (grade B).
- NIV improves sleep and quality of life in ALS patients (grade B).

### 3.12. Cognitive and behavioral symptoms

Although not conclusive, current evidence indicates that at least half of ALS patients have some form of cognitive/behavioral impairment mostly in the frontotemporal spectrum (209, 210). Large prospective studies, performed mainly on Caucasian or northern European populations, reported dementia of frontotemporal type (ALS-FTD) in approximately 15% of ALS patients while an additional 20–30% of the patients showed milder degrees of executive (ALS-eci) or non-executive (ALS-neci) cognitive impairment (210). Behavioral changes of the frontotemporal spectrum (ALS-bi) have been reported in 6–60% of patients in various studies respecting variability among populations and the assessment tools (125). There are no studies reporting detailed neuropsychological evaluation of the Iranian ALS population; however, in a Chinese study, approximately 80% of patients had normal cognition, while 11% and 5% presented ALS-eci and ALS-neci, respectively, and only 5% showed ALS-FTD (211). This lower rate of cognitive impairment compared to the Caucasian and Northern European population might in fact reflect the underlying genetic background of the Asian population. As stated above, C9ORF72 variants are the most important predictor of cognitive impairment in sporadic ALS patients and these variants are exceptionally less frequent among Eastern Asian as well as Iranian ALS patients. Therefore, a lower rate of cognitive impairment is expected in the Iranian ALS population.

Impaired cognitive function is associated with poor survival (212) and lower patient compliance to NIV and PEG (213) which necessitates proper counseling and education of the caregivers and surrogate decision-makers, especially before planning for NIV or PEG. Although the association of ALS-ci with caregiver burden is not yet known, high-quality evidence suggests that ALS-bi increases caregiver burden (214).

On the other hand, ALS-related morbidities such as respiratory failure (215) could also affect the cognitive status of ALS patients, in addition to their genetic status, such as C9orf72 repeat expansion, as discussed above (216). Therefore, it is reasonable to regularly screen for cognitive status in ALS patients starting early in the disease course to allow a more personalized care to be planned.

No specific medication or rehabilitation plan has been evaluated in ALS patients with cognitive impairment; however, memantine and cholinesterase inhibitors are usually avoided in the treatment of FTD patients without ALS due to lack of effect (217) or a possible worsening of behavioral symptoms (218). NIV might improve cognition in those with low FVC and recent onset of cognitive–behavioral impairment (219).

#### 3.12.1. Recommendations

- Regular cognitive and behavioral screening started early in the course of the disease and repeated every 6 months is strongly recommended in all ALS patients (grade A).
- Caregivers of ALS patients with behavioral impairment should receive consult and education about the possible burden of care and implications for NIV or PEG (grade B).
- Cholinesterase inhibitors or memantine are not effective in ALS patients with cognitive impairment (grade B).
- NIV treatment might improve early cognitive impairment and is recommended for ALS patients with concomitant onset of cognitive and respiratory impairments (grade B).

### 3.13. Delivering the diagnosis and discussing the prognosis

In view of the progressive nature and lack of curative treatment for ALS, delivering the diagnosis and discussing the prognosis with the patient is an important challenge for the neurologist. This information should be provided to the patient in an empathetic way and with an emphasis on giving hope and depicting the best-expected course (220).

Starting the discussion about the prognosis should be based on the patient's request. If the patient declines to receive information about the prognosis, the neurologist may ask for permission to discuss it with their family. If the patient is unable to make a decision, the family could be inquired to see whether they are willing to know the prognosis. Sometimes, an unknown and ambiguous condition is more painful for the patient. In general, knowing about the prognosis creates a sense of control and planning for the patient and caregivers (221, 222).

It is important that the discussed information be appropriate to the needs and preferences of the patient and in compliance with their beliefs and cultural background. Some patients request more detailed information, while others might only want to know general information (221). It should be emphasized to the patient and caregivers that the disease course is variable, and although it is a serious disease, many patients might follow a less aggressive course (136).

#### 3.13.1. Recommendations

- Prognosis should not be discussed after delivering the diagnosis unless specifically requested by the patient (grade B).
- If the patient wants to know the prognosis, the requested information should be provided emphasizing that the prognosis of ALS is variable (grade A).
- If the patient's preference is not to know the prognosis, upon acquiring their permission, the prognosis should be discussed with the family (grade B).
- When a patient loses decisional capacity, the physician should inquire the family about their preference for knowing the prognosis (grade B).
- In selected patients after delivering the diagnosis, a neurologist or psychiatrist might consider referral to a psychologist for further emotional care and support (grade A).

### 3.14. Depression and anxiety

Of all ALS patients, up to 44% experience depression, and up to 33% experience anxiety (223). Depression and anxiety are challenging problems that affect the quality of life and prognosis. Psychological stress is associated with decreased survival in these patients (223). Therefore, early and accurate detection and proper management of depression and anxiety in ALS patients is important and can improve the patients' quality of life. Valid clinical scales are available for screening depression and anxiety in ALS patients; however, patients should subsequently be referred to a psychiatrist for full evaluation and follow-up (224).

Recommended non-pharmacological approaches for the management of both depression and anxiety in ALS include cognitive–behavioral therapy and mindfulness techniques (224–226). Psychologists play an important role in providing these types of treatments. Pharmacological treatment of depression may include selective serotonin reuptake inhibitors, serotonin and norepinephrine reuptake inhibitors, tricyclic antidepressants, and mirtazapine based on the patient's disease status, other accompanying symptoms, and side effect profiles. selective serotonin reuptake inhibitors, bupropion, and benzodiazepines (with careful consideration of the respiratory status) should be considered for the treatment of anxiety.

#### 3.14.1. Recommendations

- Treatment of depression and anxiety in ALS patients reduces disease burden and improves their quality of life; therefore, they should be regularly screened for signs and symptoms of depression and anxiety and referred appropriately (grade A).
- Choice of an antidepressant depends on symptoms, comorbidities, and the side effect profile of the drug (grade A).
- Antidepressants such as selective serotonin reuptake inhibitors and benzodiazepine are recommended for the treatment of anxiety in ALS patients (grade A).
- Non-pharmacological treatment modalities such as cognitive–behavioral therapy and mindfulness techniques are recommended for the treatment of depression and anxiety (grade B).

### 3.15. Caregiver education and support

ALS patients are heavily reliant on the physical and spiritual support they receive from their caregivers. As a result, regular assessment of caregiver wellbeing and providing them with the support they need will have a considerable effect on the quality of life of both patients and caregivers (227). Caregivers of ALS patients with advanced disease may work up to 15 h a day and overlook their own physical and psychological health needs (228). Higher degrees of behavioral and physical impairment in ALS patients as well as caring for a tracheostomy-ventilated patient are associated with significantly increased caregiver burden (214, 229). Many caregivers experience depression and/or anxiety, while they provide care for an ALS patient (230). Psychotherapy interventions could be helpful in decreasing the psychological distress of caregivers, although evidence is currently insufficient.

#### 3.15.1. Recommendations

- Providing training and support for caregivers should be a part of ALS management (grade B).
- Formal caregiver training is especially necessary for dealing with ALS symptoms, such as impaired communication and respiratory problems, and the use of home devices such as enteral nutrition, assisted ventilation, and cough-assist devices (grade C).
- Proper counseling and psychiatry referral should be considered in high-burdened caregivers (grade B).

### 3.16. Palliative and end-of-life care; decision for invasive ventilation

Tracheostomy ventilation can prolong the life of severely disabled ALS patients for many years (231); however, it is important to consider the patient's and caregiver's quality of life, side effects, costs, and patient's desire while making the decision about tracheostomy. Although it is better to decide and perform this procedure before acute respiratory failure occurs, an Italian study showed that the decision is often made in an emergency situation, and only 30% of patients voluntarily choose tracheostomy (232). There is no universal answer for the benefit and harm balance of tracheostomy ventilation and its effect on the survival and quality of life of both patients and caregivers (233).

In a U.S. study, patients with younger age, higher socioeconomic and educational level, and those who had younger children were more inclined to seek tracheostomy and long-term mechanical ventilation (234). However, characteristics of the healthcare system, unique patient culture and religious beliefs, and country-specific regulations are other more important factors that influence patients' decision about long-term mechanical ventilation. This emphasizes the need for more local studies in this area to inform this decision (235). It should be noted that although tracheostomy prolongs life, it does not affect disease progression and does not change the final outcome (236).

Iran law does not recognize active or passive euthanasia. Moreover, there are no clear regulations about the ordering of do-not-resuscitate (DNR) code even if the patient's advanced directive is available, which is not usually the case. In specific situations involving pre-terminal ALS patients, the decision to order DNR will be upon the treating physicians.

#### 3.16.1. Recommendations

- The tracheostomy option should be discussed with all patients who have respiratory and/or bulbar weakness or are on NIV well before they reach an emergency stage that makes endotracheal intubation inevitable. This will give patients and caregivers enough time to think and decide voluntarily (grade B).
- Early tracheostomy has no effect on disease progression and significantly impairs caregivers' quality of life (grade A).

### 3.17. Decisional capacity

Patients' decisions about treatment options are especially important in ALS; therefore, it is essential for healthcare providers to provide patients with all the information they need in this process. Cognitive impairment, sometimes in the range of dementia, is an important determining factor of decisional capacity in patients with ALS. In addition, older age and lower educational level are also important contributors to impaired decisional capacity. Therefore, regular evaluation of cognitive function and decisional capacity is necessary whenever critical decisions need to be made or during treatment. For this purpose, the ECAS scale could be used to assess the patient's cognitive–behavioral status. In patients with impaired decisional capacity, the patient's previous wishes, when available, should be considered or the decision of a surrogate decision-maker should be sought. It is advisable to ask patients to choose a surrogate decision-maker while they still have the decisional capacity and keep the consent document in their health records for later reference.

#### 3.17.1. Recommendations

- In all ALS patients, it is recommended to evaluate decision-making capacity every 6 months using the ECAS as a reliable cognitive screen (grade E).
- It is recommended to seek the patient's opinion about important foreseeable future decisions, such as tracheostomy and life-sustaining measures before they lose their decision-making capacity. Otherwise, the patient should be guided to choose their surrogate in an advanced directive (grade E).

### 3.18. Organ donation

The patient's willingness to discontinue life-prolonging treatments voluntarily helps optimize opportunities for organ donation in patients with chronic neurologic diseases. A living-donor transplant from ALS patients is not directly banned in Iran; however, considering the specific situations of these patients, a more thorough physical and psychological evaluation is necessary if an ALS patient wishes to donate their organs. Depression and decisional capacity should specifically be evaluated, and it is advisable to give volunteers a mandatory decision period to be able to reevaluate their decision. It is the neurologist's responsibility to guide and assist ALS patients and caregivers in making this decision. Upon the patient's request and after providing the necessary information, a record of the signed voluntary organ donation form might be kept for future reference. Organ transplantation can be beneficial for the donor as it leads to a sense of self-esteem and having a meaningful life. On the other hand, related complications or pain after organ donation threatens the survival and quality of life of the ALS patient (237).

#### 3.18.1. Recommendations

- Approval of living-donor transplantation from ALS patients could only occur after careful cognitive and psychiatric evaluation to exclude incapacity or depression. Possible complications should be discussed with patients and caregivers (grade E).

### 3.19. Voluntary withholding or withdrawing life-sustaining treatments

Refusal of treatment is well recognized as a patient right by the Iranian “Patient's Right Charter” provided that it does not cause harm to another person and it cannot be considered a suicide attempt (238). Yet, such a decision may face physicians with a dilemma when obtaining consent for various therapeutic interventions. Setting the legal aspects aside, the first step in facing such requests is to make sure that the patient is informed of all their treatment options, the respective benefits and harms of each option, and the consequences of treatment refusal. Informed rational refusal of life-sustaining treatments by a patient with intact decisional capacity who can clearly communicate his/her reason for this choice is not considered a suicide attempt and should be respected by the treating physician (239).

Nevertheless, other regulatory, religious, and cultural aspects can affect the practice of this right by Iranian ALS patients and the response of care providers. From a Shiite perspective, although a patient's autonomy is respected, harming one's own life is not justified (240). In terminally ill patients, if demise of the patient occurs due to an act of commission by the physician (e.g., administration of a lethal substance, or turning off a ventilator), the physician is responsible to pay a “Diya,” i.e., a crime compensation. However, if death occurs due to an act of omission (e.g., refraining from intratracheal intubation, CPR, or performing a surgery such as tracheostomy), according to the contrary concept of Article 273 of the Islamic Penal Code, the physician bears no responsibility (241). Therefore, it is important for care providers to be aware of the relevant regulations and patients' rights when consulting patients who wish to refuse life-sustaining treatments.

#### 3.19.1. Recommendations

- In facing treatment refusal from an ALS patient, physicians should evaluate and document decisional capacity, inquire about suicidal ideation, and ensure that he/she is informed of all treatment options and benefits and harms of each option as well as the consequences of treatment refusal (grade E).
- Physicians should respect the choice of the competent non-suicidal ALS patient in refusing life-sustaining treatments; however, they should be aware that active facilitation in achieving patient's death is prohibited by Iran law (grade E).
- Patient refusal of life-sustaining treatments should not affect the continuation of other symptomatic treatments (grade E).

## 4. Conclusion

ALS is a progressive and disabling disease, leading to death in a few years. Although there is yet no cure for the disease, four approved disease-modifying treatments are now available that can slow disease progression. More importantly, the management of patients in multidisciplinary ALS clinics focusing on nutritional status, respiratory dysfunction, and rehabilitation improves the quality of life, thereby increasing survival. Symptomatic treatments should not be overlooked since these treatments are very helpful to control distressing symptoms and improve patients' quality of life. Management of ALS as a chronic multi-faceted disease is very nuanced and is particularly affected by cultural and social diversity as well as regional health system variabilities. In the present Iranian ALS clinical practice guideline, we tried to summarize the available research evidence in the management of various aspects of the disease, taking into consideration the related diversities, limitations, and Iranian health system characteristics.

## Author contributions

NO and RB prepared and edited the manuscript draft. RB and SN conceptualized and organized the project and revised the manuscript. All authors contributed to drafting sections, revising the manuscript, read, and approved the final version of the manuscript.
